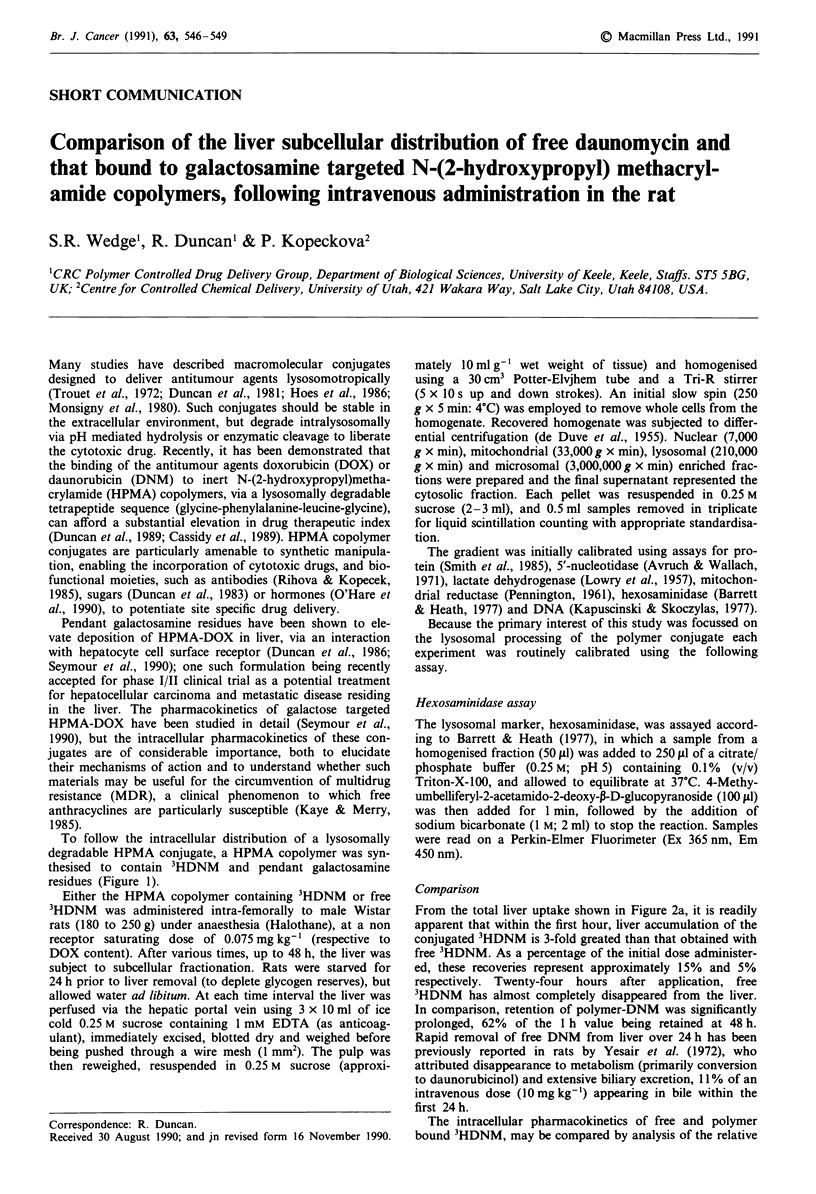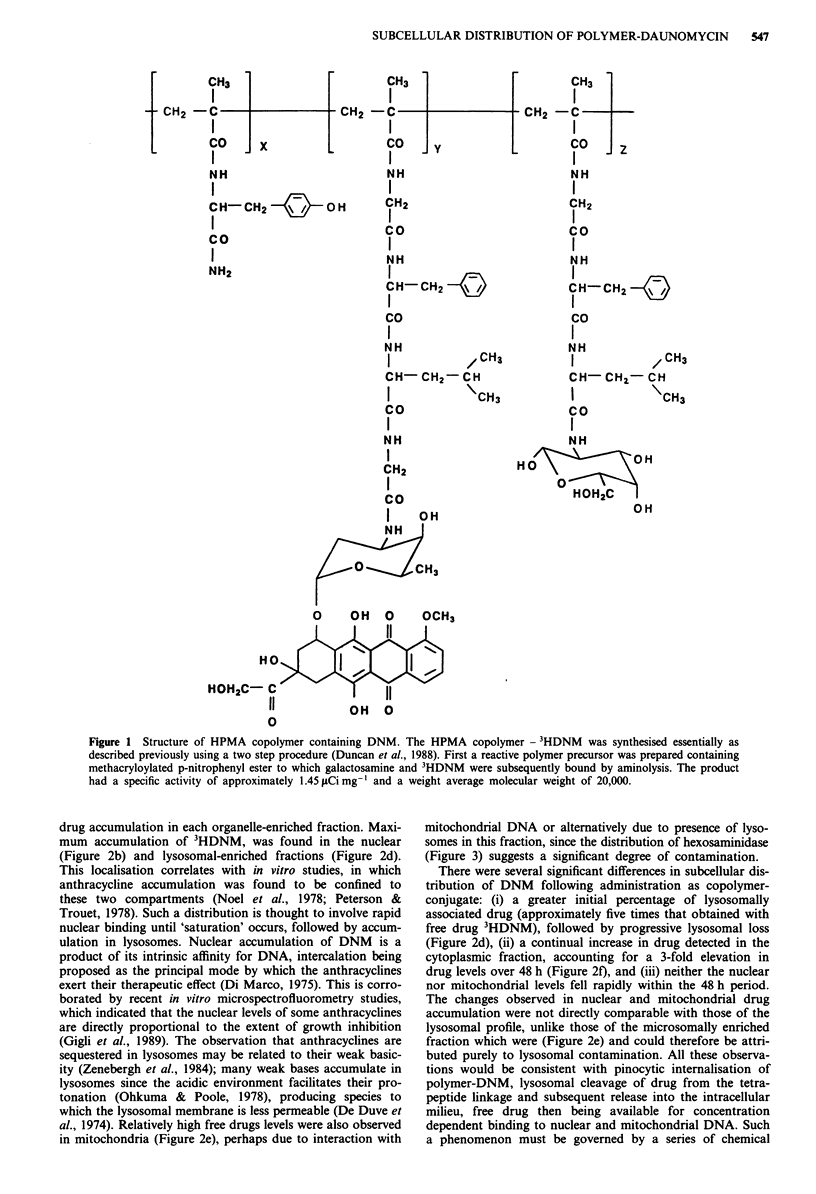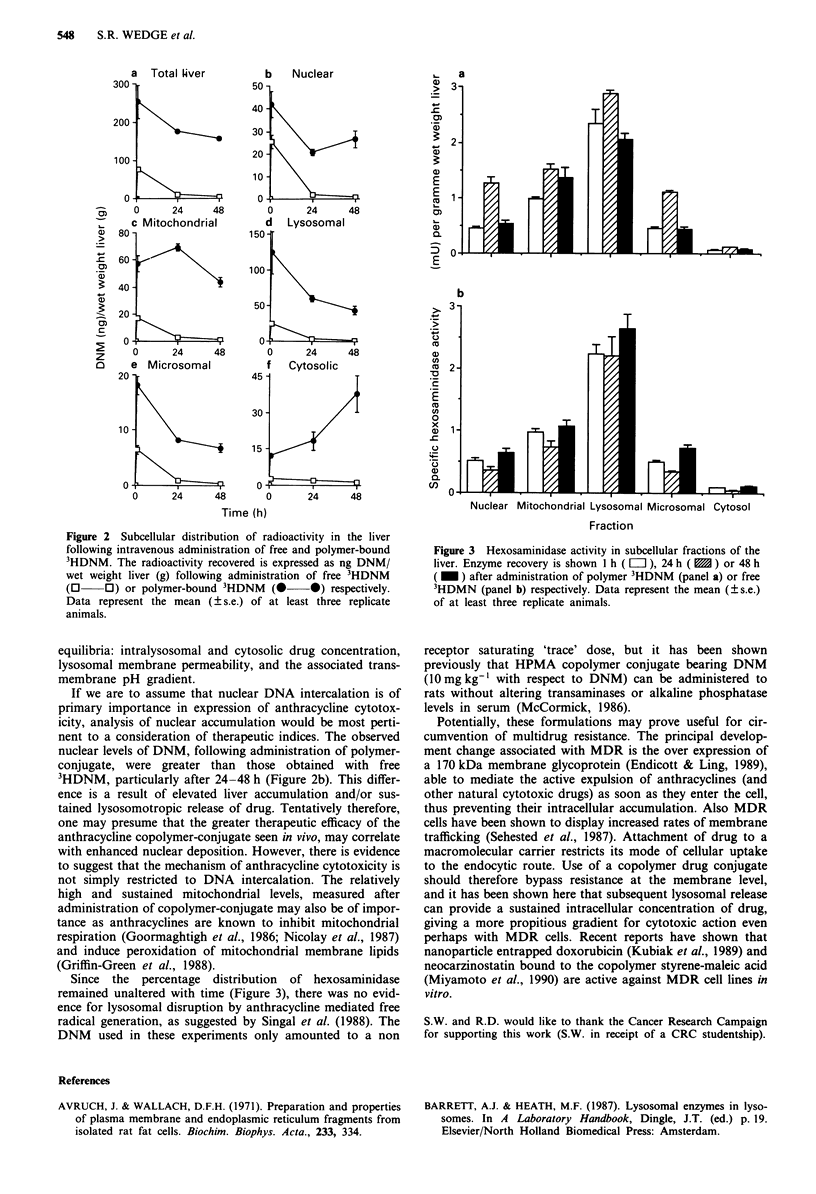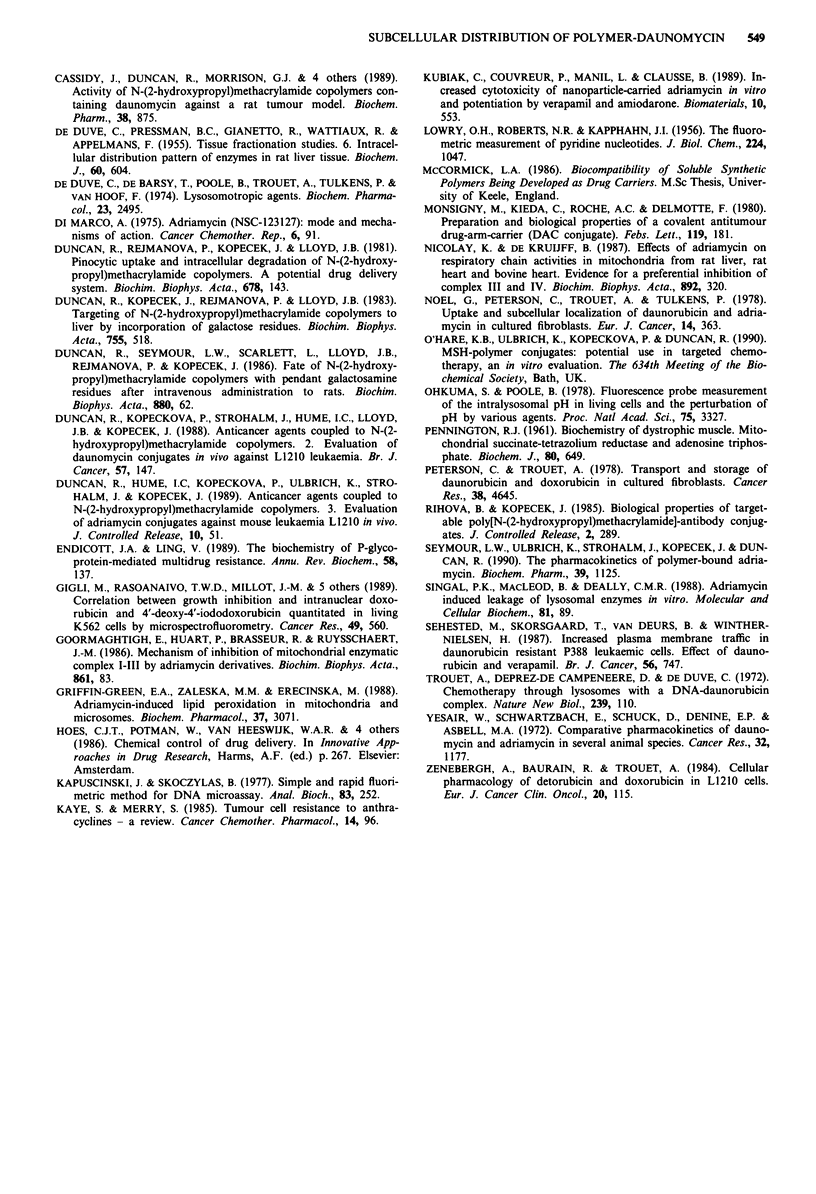# Comparison of the liver subcellular distribution of free daunomycin and that bound to galactosamine targeted N-(2-hydroxypropyl)methacrylamide copolymers, following intravenous administration in the rat.

**DOI:** 10.1038/bjc.1991.128

**Published:** 1991-04

**Authors:** S. R. Wedge, R. Duncan, P. Kopeckova

**Affiliations:** Department of Biological Sciences, University of Keele, UK.


Br. J. Cancer (1991), 63, 546–549 © Macmillan Press Ltd., 1991

SHORT COMMUNICATION

### Comparison of the liver subcellular distribution of free daunomycin and that bound to galactosamine targeted N-(2-hydroxypropyl) methacrylamide copolymers, following intravenous administration in the rat

S.R. Wedge[1], R. Duncan[1] & P. Kopeckova[2]

[1]*CRC Polymer Controlled Drug Delivery Group, Department of Biological Sciences, University of Keele, Keele, Staffs. ST5 5BG, UK;* [2]*Centre for Controlled Chemical Delivery, University of Utah, 421 Wakara Way, Salt Lake City, Utah 84108, USA.*

Many studies have described macromolecular conjugates designed to deliver antitumour agents lysosomotropically (Trouet *et al.*, 1972; Duncan *et al.*, 1981; Hoes *et al.*, 1986; Monsigny *et al.*, 1980). Such conjugates should be stable in the extracellular environment, but degrade intralysosomally via pH mediated hydrolysis or enzymatic cleavage to liberate the cytotoxic drug. Recently, it has been demonstrated that the binding of the antitumour agents doxorubicin (DOX) or daunorubicin (DNM) to inert N-(2-hydroxypropyl)methacrylamide (HPMA) copolymers, via a lysosomally degradable tetrapeptide sequence (glycine-phenylalanine-leucine-glycine), can afford a substantial elevation in drug therapeutic index (Duncan *et al.*, 1989; Cassidy *et al.*, 1989). HPMA copolymer conjugates are particularly amenable to synthetic manipulation, enabling the incorporation of cytotoxic drugs, and biofunctional moieties, such as antibodies (Rihova & Kopecek, 1985), sugars (Duncan *et al.*, 1983) or hormones (O'Hare *et al.*, 1990), to potentiate site specific drug delivery.

Pendant galactosamine residues have been shown to elevate deposition of HPMA-DOX in liver, via an interaction with hepatocyte cell surface receptor (Duncan *et al.*, 1986; Seymour *et al.*, 1990); one such formulation being recently accepted for phase I/II clinical trial as a potential treatment for hepatocellular carcinoma and metastatic disease residing in the liver. The pharmacokinetics of galactose targeted HPMA-DOX have been studied in detail (Seymour *et al.*, 1990), but the intracellular pharmacokinetics of these conjugates are of considerable importance, both to elucidate their mechanisms of action and to understand whether such materials may be useful for the circumvention of multidrug resistance (MDR), a clinical phenomenon to which free anthracyclines are particularly susceptible (Kaye & Merry, 1985).

To follow the intracellular distribution of a lysosomally degradable HPMA conjugate, a HPMA copolymer was synthesised to contain $^3$HDNM and pendant galactosamine residues (Figure 1).

Either the HPMA copolymer containing $^3$HDNM or free $^3$HDNM was administered intra-femorally to male Wistar rats (180 to 250 g) under anaesthesia (Halothane), at a non receptor saturating dose of 0.075 mg kg$^{-1}$ (respective to DOX content). After various times, up to 48 h, the liver was subject to subcellular fractionation. Rats were starved for 24 h prior to liver removal (to deplete glycogen reserves), but allowed water *ad libitum*. At each time interval the liver was perfused via the hepatic portal vein using 3 × 10 ml of ice cold 0.25 M sucrose containing 1 mM EDTA (as anticoagulant), immediately excised, blotted dry and weighed before being pushed through a wire mesh (1 mm$^2$). The pulp was then reweighed, resuspended in 0.25 M sucrose (approximately 10 ml g$^{-1}$ wet weight of tissue) and homogenised using a 30 cm$^3$ Potter-Elvjhem tube and a Tri-R stirrer (5 × 10 s up and down strokes). An initial slow spin (250 *g* × 5 min: 4°C) was employed to remove whole cells from the homogenate. Recovered homogenate was subjected to differential centrifugation (de Duve *et al.*, 1955). Nuclear (7,000 *g* × min), mitochondrial (33,000 *g* × min), lysosomal (210,000 *g* × min) and microsomal (3,000,000 *g* × min) enriched fractions were prepared and the final supernatant represented the cytosolic fraction. Each pellet was resuspended in 0.25 M sucrose (2–3 ml), and 0.5 ml samples removed in triplicate for liquid scintillation counting with appropriate standardisation.

The gradient was initially calibrated using assays for protein (Smith *et al.*, 1985), 5′-nucleotidase (Avruch & Wallach, 1971), lactate dehydrogenase (Lowry *et al.*, 1957), mitochondrial reductase (Pennington, 1961), hexosaminidase (Barrett & Heath, 1977) and DNA (Kapuscinski & Skoczylas, 1977).

Because the primary interest of this study was focussed on the lysosomal processing of the polymer conjugate each experiment was routinely calibrated using the following assay.

### *Hexosaminidase assay*

The lysosomal marker, hexosaminidase, was assayed according to Barrett & Heath (1977), in which a sample from a homogenised fraction (50 µl) was added to 250 µl of a citrate/phosphate buffer (0.25 M; pH 5) containing 0.1% (v/v) Triton-X-100, and allowed to equilibrate at 37°C. 4-Methyumbelliferyl-2-acetamido-2-deoxy-β-D-glucopyranoside (100 µl) was then added for 1 min, followed by the addition of sodium bicarbonate (1 M; 2 ml) to stop the reaction. Samples were read on a Perkin-Elmer Fluorimeter (Ex 365 nm, Em 450 nm).

### *Comparison*

From the total liver uptake shown in Figure 2a, it is readily apparent that within the first hour, liver accumulation of the conjugated $^3$HDNM is 3-fold greated than that obtained with free $^3$HDNM. As a percentage of the initial dose administered, these recoveries represent approximately 15% and 5% respectively. Twenty-four hours after application, free $^3$HDNM has almost completely disappeared from the liver. In comparison, retention of polymer-DNM was significantly prolonged, 62% of the 1 h value being retained at 48 h. Rapid removal of free DNM from liver over 24 h has been previously reported in rats by Yesair *et al.* (1972), who attributed disappearance to metabolism (primarily conversion to daunorubicinol) and extensive biliary excretion, 11% of an intravenous dose (10 mg kg$^{-1}$) appearing in bile within the first 24 h.

The intracellular pharmacokinetics of free and polymer bound $^3$HDNM, may be compared by analysis of the relative

Correspondence: R. Duncan.
Received 30 August 1990; and in revised form 16 November 1990.

**Figure 1** Structure of HPMA copolymer containing DNM. The HPMA copolymer – $^{3}$HDNM was synthesised essentially as described previously using a two step procedure (Duncan *et al.*, 1988). First a reactive polymer precursor was prepared containing methacryloylated p-nitrophenyl ester to which galactosamine and $^{3}$HDNM were subsequently bound by aminolysis. The product had a specific activity of approximately 1.45 $\mu$Ci mg$^{-1}$ and a weight average molecular weight of 20,000.

drug accumulation in each organelle-enriched fraction. Maximum accumulation of $^{3}$HDNM, was found in the nuclear (Figure 2b) and lysosomal-enriched fractions (Figure 2d). This localisation correlates with *in vitro* studies, in which anthracycline accumulation was found to be confined to these two compartments (Noel *et al.*, 1978; Peterson & Trouet, 1978). Such a distribution is thought to involve rapid nuclear binding until 'saturation' occurs, followed by accumulation in lysosomes. Nuclear accumulation of DNM is a product of its intrinsic affinity for DNA, intercalation being proposed as the principal mode by which the anthracyclines exert their therapeutic effect (Di Marco, 1975). This is corroborated by recent *in vitro* microspectrofluorometry studies, which indicated that the nuclear levels of some anthracyclines are directly proportional to the extent of growth inhibition (Gigli *et al.*, 1989). The observation that anthracyclines are sequestered in lysosomes may be related to their weak basicity (Zenebergh *et al.*, 1984); many weak bases accumulate in lysosomes since the acidic environment facilitates their protonation (Ohkuma & Poole, 1978), producing species to which the lysosomal membrane is less permeable (De Duve *et al.*, 1974). Relatively high free drugs levels were also observed in mitochondria (Figure 2e), perhaps due to interaction with mitochondrial DNA or alternatively due to presence of lysosomes in this fraction, since the distribution of hexosaminidase (Figure 3) suggests a significant degree of contamination.

There were several significant differences in subcellular distribution of DNM following administration as copolymer-conjugate: (i) a greater initial percentage of lysosomally associated drug (approximately five times that obtained with free drug $^{3}$HDNM), followed by progressive lysosomal loss (Figure 2d), (ii) a continual increase in drug detected in the cytoplasmic fraction, accounting for a 3-fold elevation in drug levels over 48 h (Figure 2f), and (iii) neither the nuclear nor mitochondrial levels fell rapidly within the 48 h period. The changes observed in nuclear and mitochondrial drug accumulation were not directly comparable with those of the lysosomal profile, unlike those of the microsomally enriched fraction which were (Figure 2e) and could therefore be attributed purely to lysosomal contamination. All these observations would be consistent with pinocytic internalisation of polymer-DNM, lysosomal cleavage of drug from the tetrapeptide linkage and subsequent release into the intracellular milieu, free drug then being available for concentration dependent binding to nuclear and mitochondrial DNA. Such a phenomenon must be governed by a series of chemical

**Figure 2** Subcellular distribution of radioactivity in the liver following intravenous administration of free and polymer-bound $^3$HDNM. The radioactivity recovered is expressed as ng DNM/wet weight liver (g) following administration of free $^3$HDNM (□——□) or polymer-bound $^3$HDNM (●——●) respectively. Data represent the mean (±s.e.) of at least three replicate animals.

**Figure 3** Hexosaminidase activity in subcellular fractions of the liver. Enzyme recovery is shown 1 h (□), 24 h (▨) or 48 h (■) after administration of polymer $^3$HDNM (panel **a**) or free $^3$HDMN (panel **b**) respectively. Data represent the mean (±s.e.) of at least three replicate animals.

equilibria: intralysosomal and cytosolic drug concentration, lysosomal membrane permeability, and the associated trans-membrane pH gradient.

If we are to assume that nuclear DNA intercalation is of primary importance in expression of anthracycline cytotoxicity, analysis of nuclear accumulation would be most pertinent to a consideration of therapeutic indices. The observed nuclear levels of DNM, following administration of polymer-conjugate, were greater than those obtained with free $^3$HDNM, particularly after 24–48 h (Figure 2b). This difference is a result of elevated liver accumulation and/or sustained lysosomotropic release of drug. Tentatively therefore, one may presume that the greater therapeutic efficacy of the anthracycline copolymer-conjugate seen *in vivo*, may correlate with enhanced nuclear deposition. However, there is evidence to suggest that the mechanism of anthracycline cytotoxicity is not simply restricted to DNA intercalation. The relatively high and sustained mitochondrial levels, measured after administration of copolymer-conjugate may also be of importance as anthracyclines are known to inhibit mitochondrial respiration (Goormaghtigh *et al.*, 1986; Nicolay *et al.*, 1987) and induce peroxidation of mitochondrial membrane lipids (Griffin-Green *et al.*, 1988).

Since the percentage distribution of hexosaminidase remained unaltered with time (Figure 3), there was no evidence for lysosomal disruption by anthracycline mediated free radical generation, as suggested by Singal *et al.* (1988). The DNM used in these experiments only amounted to a non receptor saturating 'trace' dose, but it has been shown previously that HPMA copolymer conjugate bearing DNM (10 mg kg$^{-1}$ with respect to DNM) can be administered to rats without altering transaminases or alkaline phosphatase levels in serum (McCormick, 1986).

Potentially, these formulations may prove useful for circumvention of multidrug resistance. The principal development change associated with MDR is the over expression of a 170 kDa membrane glycoprotein (Endicott & Ling, 1989), able to mediate the active expulsion of anthracyclines (and other natural cytotoxic drugs) as soon as they enter the cell, thus preventing their intracellular accumulation. Also MDR cells have been shown to display increased rates of membrane trafficking (Sehested *et al.*, 1987). Attachment of drug to a macromolecular carrier restricts its mode of cellular uptake to the endocytic route. Use of a copolymer drug conjugate should therefore bypass resistance at the membrane level, and it has been shown here that subsequent lysosomal release can provide a sustained intracellular concentration of drug, giving a more propitious gradient for cytotoxic action even perhaps with MDR cells. Recent reports have shown that nanoparticle entrapped doxorubicin (Kubiak *et al.*, 1989) and neocarzinostatin bound to the copolymer styrene-maleic acid (Miyamoto *et al.*, 1990) are active against MDR cell lines *in vitro*.

S.W. and R.D. would like to thank the Cancer Research Campaign for supporting this work (S.W. in receipt of a CRC studentship).

### References

AVRUCH, J. & WALLACH, D.F.H. (1971). Preparation and properties of plasma membrane and endoplasmic reticulum fragments from isolated rat fat cells. *Biochim. Biophys. Acta.*, **233**, 334.

BARRETT, A.J. & HEATH, M.F. (1987). Lysosomal enzymes in lysosomes. In *A Laboratory Handbook*, Dingle, J.T. (ed.) p. 19. Elsevier/North Holland Biomedical Press: Amsterdam.

CASSIDY, J., DUNCAN, R., MORRISON, G.J. & 4 others (1989). Activity of N-(2-hydroxypropyl)methacrylamide copolymers containing daunomycin against a rat tumour model. *Biochem. Pharm.*, **38**, 875.

DE DUVE, C., PRESSMAN, B.C., GIANETTO, R., WATTIAUX, R. & APPELMANS, F. (1955). Tissue fractionation studies. 6. Intracellular distribution pattern of enzymes in rat liver tissue. *Biochem. J.*, **60**, 604.

DE DUVE, C., DE BARSY, T., POOLE, B., TROUET, A., TULKENS, P. & VAN HOOF, F. (1974). Lysosomotropic agents. *Biochem. Pharmacol.*, **23**, 2495.

DI MARCO, A. (1975). Adriamycin (NSC-123127): mode and mechanisms of action. *Cancer Chemother. Rep.*, **6**, 91.

DUNCAN, R., REJMANOVA, P., KOPECEK, J. & LLOYD, J.B. (1981). Pinocytic uptake and intracellular degradation of N-(2-hydroxypropyl)methacrylamide copolymers. A potential drug delivery system. *Biochim. Biophys. Acta.*, **678**, 143.

DUNCAN, R., KOPECEK, J., REJMANOVA, P. & LLOYD, J.B. (1983). Targeting of N-(2-hydroxypropyl)methacrylamide copolymers to liver by incorporation of galactose residues. *Biochim. Biophys. Acta.*, **755**, 518.

DUNCAN, R., SEYMOUR, L.W., SCARLETT, L., LLOYD, J.B., REJMANOVA, P. & KOPECEK, J. (1986). Fate of N-(2-hydroxypropyl)methacrylamide copolymers with pendant galactosamine residues after intravenous administration to rats. *Biochim. Biophys. Acta.*, **880**, 62.

DUNCAN, R., KOPECKOVA, P., STROHALM, J., HUME, I.C., LLOYD, J.B. & KOPECEK, J. (1988). Anticancer agents coupled to N-(2-hydroxypropyl)methacrylamide copolymers. 2. Evaluation of daunomycin conjugates *in vivo* against L1210 leukaemia. *Br. J. Cancer*, **57**, 147.

DUNCAN, R., HUME, I.C, KOPECKOVA, P., ULBRICH, K., STROHALM, J. & KOPECEK, J. (1989). Anticancer agents coupled to N-(2-hydroxypropyl)methacrylamide copolymers. 3. Evaluation of adriamycin conjugates against mouse leukaemia L1210 *in vivo*. *J. Controlled Release*, **10**, 51.

ENDICOTT, J.A. & LING, V. (1989). The biochemistry of P-glycoprotein-mediated multidrug resistance. *Annu. Rev. Biochem.*, **58**, 137.

GIGLI, M., RASOANAIVO, T.W.D., MILLOT, J.-M. & 5 others (1989). Correlation between growth inhibition and intranuclear doxorubicin and 4'-deoxy-4'-iododoxorubicin quantitated in living K562 cells by microspectrofluorometry. *Cancer Res.*, **49**, 560.

GOORMAGHTIGH, E., HUART, P., BRASSEUR, R. & RUYSSCHAERT, J.-M. (1986). Mechanism of inhibition of mitochondrial enzymatic complex I-III by adriamycin derivatives. *Biochim. Biophys. Acta.*, **861**, 83.

GRIFFIN-GREEN, E.A., ZALESKA, M.M. & ERECINSKA, M. (1988). Adriamycin-induced lipid peroxidation in mitochondria and microsomes. *Biochem. Pharmacol.*, **37**, 3071.

HOES, C.J.T., POTMAN, W., VAN HEESWIJK, W.A.R. & 4 others (1986). Chemical control of drug delivery. In *Innovative Approaches in Drug Research*, Harms, A.F. (ed.) p. 267. Elsevier: Amsterdam.

KAPUSCINSKI, J. & SKOCZYLAS, B. (1977). Simple and rapid fluorimetric method for DNA microassay. *Anal. Bioch.*, **83**, 252.

KAYE, S. & MERRY, S. (1985). Tumour cell resistance to anthracyclines - a review. *Cancer Chemother. Pharmacol.*, **14**, 96.

KUBIAK, C., COUVREUR, P., MANIL, L. & CLAUSSE, B. (1989). Increased cytotoxicity of nanoparticle-carried adriamycin *in vitro* and potentiation by verapamil and amiodarone. *Biomaterials*, **10**, 553.

LOWRY, O.H., ROBERTS, N.R. & KAPPHAHN, J.I. (1956). The fluorometric measurement of pyridine nucleotides. *J. Biol. Chem.*, **224**, 1047.

MCCORMICK, L.A. (1986). *Biocompatibility of Soluble Synthetic Polymers Being Developed as Drug Carriers.* M.Sc Thesis, University of Keele, England.

MONSIGNY, M., KIEDA, C., ROCHE, A.C. & DELMOTTE, F. (1980). Preparation and biological properties of a covalent antitumour drug-arm-carrier (DAC conjugate). *Febs. Lett.*, **119**, 181.

NICOLAY, K. & DE KRUIJFF, B. (1987). Effects of adriamycin on respiratory chain activities in mitochondria from rat liver, rat heart and bovine heart. Evidence for a preferential inhibition of complex III and IV. *Biochim. Biophys. Acta.*, **892**, 320.

NOEL, G., PETERSON, C., TROUET, A. & TULKENS, P. (1978). Uptake and subcellular localization of daunorubicin and adriamycin in cultured fibroblasts. *Eur. J. Cancer*, **14**, 363.

O'HARE, K.B., ULBRICH, K., KOPECKOVA, P. & DUNCAN, R. (1990). MSH-polymer conjugates: potential use in targeted chemotherapy, an *in vitro* evaluation. *The 634th Meeting of the Biochemical Society*, Bath, UK.

OHKUMA, S. & POOLE, B. (1978). Fluorescence probe measurement of the intralysosomal pH in living cells and the perturbation of pH by various agents. *Proc. Natl Acad. Sci.*, **75**, 3327.

PENNINGTON, R.J. (1961). Biochemistry of dystrophic muscle. Mitochondrial succinate-tetrazolium reductase and adenosine triphosphate. *Biochem. J.*, **80**, 649.

PETERSON, C. & TROUET, A. (1978). Transport and storage of daunorubicin and doxorubicin in cultured fibroblasts. *Cancer Res.*, **38**, 4645.

RIHOVA, B. & KOPECEK, J. (1985). Biological properties of targetable poly[N-(2-hydroxypropyl)methacrylamide]-antibody conjugates. *J. Controlled Release*, **2**, 289.

SEYMOUR, L.W., ULBRICH, K., STROHALM, J., KOPECEK, J. & DUNCAN, R. (1990). The pharmacokinetics of polymer-bound adriamycin. *Biochem. Pharm.*, **39**, 1125.

SINGAL, P.K., MACLEOD, B. & DEALLY, C.M.R. (1988). Adriamycin induced leakage of lysosomal enzymes *in vitro*. *Molecular and Cellular Biochem.*, **81**, 89.

SEHESTED, M., SKORSGAARD, T., VAN DEURS, B. & WINTHER-NIELSEN, H. (1987). Increased plasma membrane traffic in daunorubicin resistant P388 leukaemic cells. Effect of daunorubicin and verapamil. *Br. J. Cancer*, **56**, 747.

TROUET, A., DEPREZ-DE CAMPENEERE, D. & DE DUVE, C. (1972). Chemotherapy through lysosomes with a DNA-daunorubicin complex. *Nature New Biol.*, **239**, 110.

YESAIR, W., SCHWARTZBACH, E., SCHUCK, D., DENINE, E.P. & ASBELL, M.A. (1972). Comparative pharmacokinetics of daunomycin and adriamycin in several animal species. *Cancer Res.*, **32**, 1177.

ZENEBERGH, A., BAURAIN, R. & TROUET, A. (1984). Cellular pharmacology of detorubicin and doxorubicin in L1210 cells. *Eur. J. Cancer Clin. Oncol.*, **20**, 115.